# Continuous venovenous hemofiltration versus extended daily hemofiltration in patients with septic acute kidney injury: a retrospective cohort study

**DOI:** 10.1186/cc13827

**Published:** 2014-04-09

**Authors:** Zhiping Sun, Hong Ye, Xia Shen, Hongdi Chao, Xiaochun Wu, Junwei Yang

**Affiliations:** 1 Center of Kidney Disease, Second Affiliated Hospital, Nanjing Medical University, 262 North Zhongshan Road, Jiangsu Province, Nanjing 210003, China

## Abstract

**Introduction:**

Whether continuous venovenous hemofiltration (CVVHF) is superior to extended daily hemofiltration (EDHF) for the treatment of septic AKI is unknown. We compared the effect of CVVHF (greater than 72 hours) with EDHF (8 to 12 hours daily) on renal recovery and mortality in patients with severe sepsis or septic shock and concurrent acute kidney injury (AKI).

**Methods:**

A retrospective analysis of 145 septic AKI patients who underwent renal replacement therapy (RRT) between July 2009 and May 2013 was performed. These patients were treated by CVVHF or EDHF with the same polyacrylonitrile membrane and bicarbonate-based buffer. The primary outcomes measured were occurrence of renal recovery and all-cause mortality by 60 days.

**Results:**

Sixty-five and eighty patients were treated with CVVHF and EDHF, respectively. Patients in the CVVHF group had significantly higher recovery of renal function (50.77% of CVVHF group versus 32.50% in the EDHF group, *P* = 0.026). Median time to renal recovery was 17.26 days for CVVHF patients and 25.46 days for EDHF patients (*P* = 0.039). Sixty-day all-cause mortality was similar between CVVHF and EDHF groups (44.62%, and 46.25%, respectively; *P* = 0.844). 55.38% of patients on CVVHF and 28.75% on EDHF developed hypophosphatemia (*P* = 0.001). The other adverse events related to RRT did not differ between groups. On multivariate analysis, including physiologically clinical relevant variables, CVVHF therapy was significantly associated with recovery of renal function (HR 3.74; 95% CI 1.82 to 7.68; *P* < 0.001), but not with mortality (HR 0.69; 95% CI 0.34 to 1.41; *P* = 0.312).

**Conclusions:**

Patients undergoing CVVHF therapy had significantly improved renal recovery independent of clinically relevant variables. The patients with septic AKI had similar 60-day all-cause mortality rates, regardless of type of RRT.

## Introduction

Sepsis and septic shock are the most important causes of acute kidney injury (AKI), accounting for at least 50% of AKI in ICU patients. An estimated 700,000 cases of sepsis occur yearly, resulting in more than 210,000 deaths in the United States. The combination of AKI and sepsis is associated with a mortality rate of 70%, compared with 45% among patients with AKI alone [1,2]. Multiple clinical treatments, including volume resuscitation and vasoconstrictor therapy, are only marginally effective in improving renal function and reducing mortality [3].

Since the first description of continuous arteriovenous hemofiltration in 1977, continuous renal replacement therapy (CRRT) has gained widespread acceptance for the treatment of AKI in hemodynamically unstable patients [4]. As improved hemodynamics is associated with less renal ischemia, CRRT may hasten recovery of renal function, and even result in increased survival [5]. CRRT strategies include mainly continuous venovenous hemodialysis (CVVHD) and continuous venovenous hemofiltration (CVVHF) [6]. Despite a similar clearance of small molecules between the two modalities, Brunet *et al*. [7] found that CVVHF increases the clearance of medium to larger molecules compared to CVVHD. As a result, CVVHF can more effectively reduce the effects of systemic inflammatory response syndrome in critically ill patients with sepsis by clearing large toxic inflammatory cytokines, most of which are in the middle molecular-weight range. There are multiple mechanisms by which sepsis can induce AKI. The persistently elevated levels of inflammatory mediators, coupled with severe endothelial dysfunction and hemodynamic instability, may synergistically induce kidney injury [8,9]. Thus, continuous hemofiltration may mitigate or stabilize patients with AKI and sepsis, facilitating renal recovery. In several studies of CRRT in AKI, however, although delivered doses and RRT modality are mentioned, treatment time and time of interruption of treatment are not discussed [10-12]. Whether continuous hemofiltration therapy leads to improved outcomes in septic patients with AKI remains unclear.

In the study, we compared CVVHF (continuous, first treatment greater than 72 hours) with extended daily hemofiltration (EDHF, 8 to 12 hours daily) in patients with AKI from severe sepsis or septic shock, with regards to recovery of renal function, adverse events, and mortality.

## Materials and methods

### Study population

**Figure 1:**
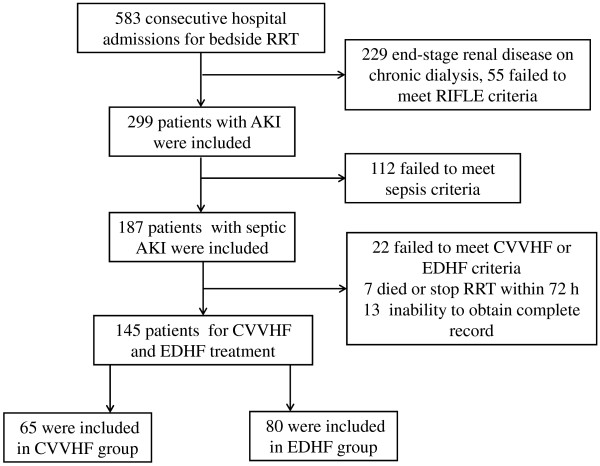
**Study profile.** RRT, renal replacement therapy; AKI, acute kidney injury; CVVHF, continuous venovenous hemofiltration; EDHF, extended daily hemofiltration; RIFLE, risk injury failure loss end-stage renal failure.

We performed a retrospective analysis of a prospectively collected cohort of consecutive adult patients (>18 years of age) who underwent RRT treatment between July 2009 and May 2013 in our institution. The diagnoses of severe sepsis and septic shock were classified according to the American College of Chest Physicians/Society of Critical Care Medicine criteria [13]. AKI was determined according to the risk, injury, failure, loss, end-stage renal failure (RIFLE) criteria [14]. RIFLE class was determined based on the highest creatinine level or lowest estimated glomerular filtration rate (eGFR) or urine output (UO) (hourly, or in 12 hours). Baseline creatinine levels (within 3 months of hospital admission) were available for most patients. In patients without baseline creatinine values, the modification of diet in renal disease (MDRD) equation as recommended by the ADQI Working Group was used (assuming a lower limit of baseline GFR of 75 ml/minute) [15]. Exclusion criteria were: non-septic AKI, failure to meet CVVHF or EDHF criteria, the death of patients or cessation of treatment within 72 hours after receiving RRT, and inability to obtain a complete medical record (Figure 1). The criterion enrolled into the CVVHF group was at least 72 hours at the first RRT treatment. Patients enrolled into the EDHF group received hemofiltration treatment for at least 8 hours, and for no more than 12 hours daily.

Demographic characteristics, comorbidities, and location of treatment (ICU, surgical or medical ward), were obtained from hospital data. Comorbid diseases were defined as: hypertension (blood pressure above 140/90 mm Hg or use of anti-hypertensive agents prior to hospitalization); diabetes mellitus (previously diagnosed, and usage of insulin or oral hypoglycemic agents); congestive heart failure (low cardiac output with a central venous pressure (CVP) above 12 mm Hg, and requirement for a dopamine equivalent at a rate of 5 μg/kg/minute or higher at the initiation of RRT) [16]; chronic kidney disease (CKD) (defined as greater documented prior to this admission). Laboratory data including white blood-cell and platelet counts, serum electrolytes (sodium and potassium), renal function (urea and creatinine), C-reactive protein (CRP), and lactate, were recorded upon initiation of RRT. Study investigators recorded respiratory rate, heart rate, temperature, arterial pressures, UO, requirement for mechanical ventilation and arterial blood gas measurements at the beginning of RRT. Acute physiologic and chronic health evaluation II (APACHE II) scores and sequential organ failure assessment (SOFA) scores were calculated to evaluate severity of illness.

### Renal replacement therapy procedures

The requirement for RRT was determined by the consulting nephrologist and was monitored by nursing staff with experience in venovenous hemofiltration. Patients received different RRT modalities mainly on the basis of different clinical situations and laboratory outcomes. Polyacrylonitrile hollow-fibre hemofilters (AEF-10; Asahi Kasei Kuraiay Medical Co. Ltd., Tokyo, Japan) with surface areas of 1.0 m^2^ or 1.3 m^2^ were used in all patients. Bicarbonate-buffered replacement fluids were used for all RRT procedures. Replacement fluids were added in the predilutional mode. The cumulative ultrafiltration rate was measured hourly from the machine display. Net ultrafiltration was set by the nephrologist in charge according to the patient’s condition and clinical need. As CVVHF treatment was performed continuously, to ensure the use of efficient membranes, the hemofilter was replaced routinely every 24 hours, and at other recorded times if the filter clotted prematurely. The extracorporeal circuit was anticoagulated by continuous unfractionated heparin, low molecular-weight heparin, or others, including citrate solutions and argatroban. Of the 65 patients in the CVVHF group, 25 (38.75%) were changed to EDHF or intermittent hemodialysis (IHD) after more than 72 hours when UO was significantly increased without cardiac dysfunction and pulmonary edema. RRT was withdrawn when urine output was greater than 1,000 ml/day with improving or normal renal function. Complications of RRT were recorded on the dialysis flow sheets as secondary outcome measures.

### Baseline assessment and data collection

The primary outcome measure was 60-day recovery of renal function and all-cause mortality. Secondary outcome measures included time of extrarenal support and occurrence of adverse events. Time to recovery of renal function was defined as the time to definitive withdrawal of RRT. Adverse events were recorded throughout all episodes, per patient, from inclusion until withdrawal of RRT. Hypotension was defined as a systolic arterial pressure of 80 mm Hg or less, or a fall greater than 50 mm Hg from the baseline value, hypophosphatemia as blood phosphate concentrations of less than 0.6 mmol/L, and hypokalemia as blood potassium concentrations of less than 3.0 mmol/L. Bleeding events necessitating transfusion were recorded. Daily fluid balance (FB) was calculated as the difference between fluid administered (intravenous fluids + blood products + enteral fluids + RRT replacement fluids) and fluid lost (urine output + blood losses + enteral losses + drain losses + replacement fluids from RRT, and net ultrafiltration rate). The mean FB and mean arterial pressure (MAP) during the first 72 hours were calculated by hourly blood pressure while on RRT. Replacement fluids, net ultrafiltration, and blood pressure were collected from recordings of dialysis flow sheets and nursing records of ICU or wards.

The retrospective study was approved by the Medical Ethics Committee of the Affiliated Second Hospital (Nanjing, China). The Committee has waived the informed consent of the analysis of retrospective data.

### Statistical analyses

All variables were expressed as the mean ± standard error of the mean (SEM) and categorical data as the actual numbers with percentages. Univariate analysis was performed to compare variables between groups, using the unpaired *t*-test for digit variables and the χ^2^ test for categorical variables. Comparison of the total fluid balance between groups was performed using the Mann-Whitney *U*-test.

The probability of overall mortality and recovery of renal function were estimated by the Kaplan-Meier method. The Cox proportional hazards model was used to identify predictors of overall mortality and recovery of renal function. For the multivariate analysis (mortality and recovery of renal function), a univariate factor with a *P*-value of less than 0.2 was used for model selection. Additionally, multivariate analysis was repeated by including all clinically relevant covariates, regardless of their significance in the univariate analysis, and CVVHF and all varieties were forced into multivariate models. Variables with a two-tailed *P*-value of less than 0.05 were considered statistically significant. All statistical analyses were performed using SPSS (SPSS Inc., Chicago, IL, USA) version 17.5 for Windows.

## Results

The study profile is shown in Figure 1: 583 patients undergoing bedside RRT were screened, 299 patients with AKI were included, 187 met criteria for septic AKI, and 145 met the inclusion criteria of CVVHF (n = 65) and EDHF treatment (n = 80). A comparison of patient characteristics, selected laboratory values, and physiologic variables at the time of initiation of RRT between groups are shown in Table 1. There were no differences in baseline and clinical characteristics between CVVHF and EDHF patients. Oliguria was more common in patients treated by CVVHF (*P* = 0.057). When classified according to the RIFLE criteria, there was no difference in risk, injury, and failure between groups, indicating that the timing of RRT initiation has no distinction in patients on CVVHF and EDHF. Baseline creatinine was available in 91% of patients. The diagnosis of AKI and RRT initiation were based on UO criteria in 29% of patients in the CVVHF group and 18% in the EDHF group. A higher proportion of patients with septic shock received CVVHF compared with EDHF, but this difference was not found to be statistically significant (*P* = 0.060). Although patients undergoing CVVHF had a lower MAP and blood pH compared with those receiving EDHF (*P* = 0.034, 0.036; respectively), the two groups were well-balanced in the APACHE II (*P* = 0.472) and SOFA organ-system scores (*P* = 0.213). There was no significant difference in median time from AKI to initiation of RRT.

**Table 1:** Patient demographics and baseline characteristics

	**CVVHF (n = 65)**	**EDHF (n = 80)**	***P*-value**
Age, years	67.78 ± 17.55	68.59 ± 14.97	0.407
Female, n (%)	16 (24.62)	21 (26.25)	0.822
Hypertension, n (%)	25 (38.46)	26 (32.50)	0.455
Diabetes mellitus, n (%)	19 (29.23)	19 (23.75)	0.455
Chronic kidney disease, n (%)	10 (15.38)	15 (18.75)	0.594
Congestive heart failure, n (%)	18 (27.69)	17 (21.25)	0.367
Mechanical ventilation, n (%)	28 (43.08)	31 (38.75)	0.598
Reason for admission			
Medical, n (%)	49 (75.39)	58 (72.50)	0.694
Surgical, n (%)	16 (24.61)	22 (27.50)	
Se-psis type			
Severe sepsis, n (%)	44 (67.69)	65 (81.25)	0.060
Septic shock, n (%)	21 (32.31)	15 (18.75)	
Classification of AKI			
Risk, n (%)	12 (18.46)	13 (16.25)	0.936
Injury, n (%)	25 (38.46)	31 (38.75)	
Failure, n (%)	28 (43.08)	36 (45.00)	
Oliguria, n (%)	48 (73.85)	47 (58.75)	0.057
Median time from AKI occurrence to initiation of RRT, days	3.32 ± 2.06	3.78 ± 2.23	0.277
MAP, mm Hg	65.18 ± 17.49	73.69 ± 17.21	0.037
Respiratory rate, breaths/minute	23.03 ± 7.08	22.94 ± 10.05	0.482
Heart rate, beats/minute	103.78 ± 19.09	99.80 ± 22.58	0.187
Temperature, °C	37.55 ± 1.47	37.40 ± 1.22	0.301
Serum urea, mmol/L	28.79 ± 16.61	32.66 ± 13.38	0.113
Serum creatinine, μmol/L	399.30 ± 240.71	443.42 ± 218.29	0.182
eGFR, ml/minute	17.89 ± 15.32	13.98 ± 13.58	0.102
PH	7.22 ± 0.14	7.27 ± 0.13	0.036
HCO_3_^-^ mmol/L	14.99 ± 5.39	16.79 ± 5.43	0.061
Lactate, meq/L	3.98 ± 1.32	3.21 ± 1.91	0.330
Serum sodium, mmol/L	137.37 ± 10.40	139.71 ± 11.45	0.159
Serum potassium, mmol/L	4.53 ± 1.13	4.49 ± 1.13	0.444
White blood-cell count, 10^9^/L	15.43 ± 7.07	14.28 ± 7.10	0.209
Platelet count, 10^9^/L	137.67 ± 80.82	147.53 ± 95.62	0.314
CRP, ng/dl	95.44 ± 66.59	85.19 ± 56.59	0.216
APACHE II score	31.10 ± 7.23	31.20 ± 7.12	0.472
SOFA organ-system score	11.98 ± 2.92	12.29 ± 3.05	0.213

AKI, acute kidney injury; RRT, renal replacement therapy; MAP, mean arterial pressure; eGFR, estimated glomerular filtration rate; HCO_3_^-^, bicarbonate; CRP, C-reactive protein; APACHE II, acute physiologic and chronic health evaluation; SOFA, sequential organ failure assessment; CVVHDF, continuous venovenous hemodiafiltration; CVVHF, continuous venovenous hemofiltration.

The details regarding RRT between groups are shown in Table 2. Mean blood flow between groups was significantly different (*P* = 0.005). Mean ultrafiltration per kilogram per hour was significantly lower in patients receiving CVVHF (149.09 ± 66.58 ml) compared with those receiving EDHF (241.84 ± 92.64 ml, *P* <0.001). However, ultrafiltration per day was higher in CVVHF versus EDHF recipients (3.20 ± 1.59 l versus 2.84 ± 1.09 l, *P* = 0.110). Total FB was markedly different between groups. The median cumulative total FB during the first 72 hours of treatment was -0.46 ± 0.67 l for the CVVHF group and 0.15 ± 0.37 l for the EDHF group (*P* = 0.019). There was no difference between groups in MAP in the first 72 hours after initiation of RRT (*P* = 0.137).

**Table 2:** RRT characteristics, complications, and outcomes by treatment group

	**CVVHF (n = 65)**	**EDHF (n = 80)**	***P *-value**
Blood flow, ml/minute	213.03 ± 21.72	231.43 ± 18.26	0.005
Ultrafiltration per hour, ml	149.09 ± 66.58	241.84 ± 92.64	<0.001
Ultrafiltration per day, L	3.20 ± 1.59	2.84 ± 1.09	0.110
Replacement flow, ml/kg/h	29.42 ± 6.10	40.14 ± 8.19	<0.001
Replacement flow, L/h	19.32 ± 5.32	26.32 ± 6.64	<0.001
Replacement flow, L/day	4.64 ± 1.28	2.65 ± 0.75	<0.001
The total time of renal support, days	3.86 ± 1.55	3.41 ± 1.58	0.112
Anticoagulant, n (%)			
None	10 (15.38)	20 (25.00)	0.355
Heparin/low molecular heparin	35 (53.85)	37 (46.25)	
Others	20 (27.69)	23 (28.75)	
Fluid balance during first 72 hours RRT, L	-0.46 ± 0.67	0.15 ± 0.37	0.019
MAP during first 72 hours RRT, mm Hg	89.73 ± 20.01	83.79 ± 19.26	0.137
Complications, n (%)			
Bleeding	14 (21.54)	10 (12.50)	0.145
Hypotension	10 (15.39)	21 (26.25)	0.112
Hypokalemia	33 (50.77)	31 (38.75)	0.147
Hypophosphatemia	36 (55.38)	23 (28.75)	0.001
Primary outcome, n (%)			
Renal recovery-60 days	33 (50.77)	26 (32.50)	0.026
Mortality-60 days	29 (44.62)	37 (46.25)	0.844

RRT, renal replacement therapy; MAP, mean arterial pressure; CVVHDF, continuous venovenous hemodiafiltration; CVVHF, continuous venovenous hemofiltration.

**Figure 2:**
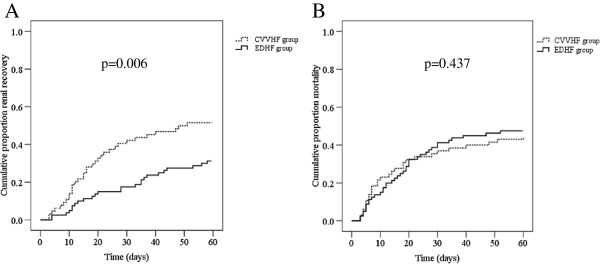
Kaplan-Meier analysis of recovery rates of renal function (A) and all-cause 60-day mortality (B) between continuous venovenous hemofiltration (CVVHF) and extended daily hemofiltration (EDHF) groups.

By Kaplan-Meier curves, we demonstrated that the proportion of renal recovery at 60 days was much higher in CVVHF group as compared with EDHF group (*P* = 0.006; Figure 2A). The recovery of renal function by 60 days was 50.77% in the CVVHF group compared with 32.50% in the EDHF group (*P* = 0.026; Table 2). Median time to renal recovery was 17.26 days for CVVHF patients (95% CI 15.89 to 19.62), and 25.46 days for EDHF patients (95% CI 18.72 to 32.19; *P* = 0.039). However, mortality was similar between two treatment groups (*P* = 0.437; Figure 2B): 29 of the 65 patients (44.62%) and 37 of the 80 patients (46.25%) undergoing CVVHF and EDHF therapy died within 60 days (*P* = 0.844; Table 2). Median time to death was similar between groups, with 17.24 days (95% CI 11.48 to 23.00) for the CVVHF group and 18.97 days for the EDHF group (95% CI 14.80 to 23.14; *P* = 0.851). We unexpectedly found that 5 of 65 patients receiving CVVHF therapy (7.69%) and 6 of 80 patients receiving EDHF (7.50%) died within 60 days (*P* = 0.965), even though they had recovered renal function. In addition, 10 of 80 (12.5%) patients in the EDHF group required intermittent dialysis, compared with 6 of 65 (9.23%) patients in the CVVHF group (*P* = 0.532).

As shown in Table 2, there was no significant difference in the total time of renal support between groups (*P* = 0.112). Of note, 55.38% of patients on CVVHF developed hypophosphatemia, compared to 28.75% on of EDHF (*P* = 0.001). The frequency of other adverse events including bleeding, hypotension and hypokalemia did not significantly differ between groups.

The Cox proportional hazard model was used to predict 60-day renal recovery and mortality. By univariate analysis, lower SOFA and APACHE II scores and higher eGFR levels correlated with improved renal recovery (Table 3). CVVHF therapy was significantly associated with recovery of renal function (hazard ratio (HR) 3.49; 95% CI 1.77 to 6.87; *P* <0.001). The lower MAP and blood pH prior to initiation of RRT trended towards less recovery of renal function (*P* = 0.078, 0.082 respectively; Table 3). After adjusting for the multivariate analysis (selected variables with a *P*-value <0.2 in the univariate analysis), lower APACHE II scores and CVVHF therapy were still significantly associated with improved renal recovery (*P* = 0.004, 0.001 respectively; Table 3). When using the Cox regression model to predict risk factors for all-cause mortality, we found that lower platelet levels, pH, and MAP, and higher SOFA and APACHE II scores were associated with a significantly higher mortality risk in the univariate analysis (Table 4). However, CVVHF therapy was not associated with mortality (HR 0.65; 95% CI 0.37 to 1.24; *P* = 0.191). In the multivariate analysis, higher SOFA scores remained significant predictors of 60-day mortality (HR 1.34; 95% CI 1.18 to 1.53; *P* <0.001), and CVVHF therapy was not associated with mortality (HR 0.80; 95% CI 0.37 to 1.72; *P* = 0.571).

**Table 3:** Independent predictors for 60-day renal recovery using the Cox proportional hazards model

	**Univariate**			**Multivariate**		
	**Hazard ratio**	**95% CI**	***P*-value**	**Hazard ratio**	**95% CI**	***P*-value**
MAP	1.02	0.98, 1.04	0.078	0.99	0.98, 1.01	0.560
PH	1.28	0.97, 1.68	0.082	1.13	0.80, 1.58	0.489
eGFR	1.02	1.01, 1.03	0.002	1.01	0.99, 1.02	0.431
APACHEII	0.88	0.83, 0.93	<0.001	0.90	0.84, 0.97	0.004
SOFA	0.80	0.71, 0.90	<0.001	0.93	0.78, 1.10	0.381
CVVHF	3.49	1.77, 6.87	<0.001	3.79	1.89, 7.85	<0.001

MAP, mean arterial pressure; eGFR, estimated glomerular filtration rate; APACHE II, acute physiologic and chronic health evaluation; SOFA, sequential organ failure assessment; CVVHF, continuous venovenous hemofiltration.

**Table 4:** Independent predictors for 60-day mortality using the Cox proportional hazards model

	**Univariate**			**Multivariate**		
	**Hazard ratio**	**95% CI**	***P*-value**	**Hazard ratio**	**95% CI**	***P*-value**
Platelet count	0.95	0.91-0.99	0.013	1.00	0.99-1.01	0.503
MAP	0.97	0.94-0.99	0.022	0.98	0.97-1.00	0.404
PH	0.67	0.54-0.85	0.001	0.81	0.63-1.03	0.088
APACHEII	1.02	1.06-1.16	<0.001	1.06	1.01-1.11	0.039
SOFA	1.44	1.28-1.62	<0.001	1.34	1.18-1.53	<0.001
CVVHF	0.65	0.34-1.24	0.191	0.80	0.37-1.72	0.571

MAP, mean arterial pressure; eGFR, estimated glomerular filtration rate; APACHE II, acute physiologic and chronic health evaluation; SOFA, sequential organ failure assessment; CVVHF, continuous venovenous hemofiltration.

The multivariate analysis was repeated including physiologically clinical relevant variables (age, gender, and comorbidities, including hypertension, diabetes mellitus, congestive heart failure, chronic kidney diseases (CKD), surgery, and septic shock), regardless of statistical significance in the univariate analysis. Similar results were obtained, specifically that CVVHF was significantly associated with the recovery of renal function (HR 3.74; 95% CI 1.82 to 7.68; *P* <0.001), but not with mortality (HR 0.69; 95% CI 0.34 to 1.41; *P* = 0.312).

## Discussion

We compared CVVHF (continuous, greater than 72 hours) with EDHF (8 to 12 hours daily) for the treatment of AKI in patients with sepsis. Due to the typically lengthy hospital stay of critically ill patients, our primary endpoint was recovery of renal function and mortality rates within 60 days. A significant difference in the incidence of and time to recovery of renal function was found. Patients undergoing CVVHF therapy had a higher incidence of and shorter time to renal recovery. However, there were no significant differences in mortality: 44.62% and 46.25% patients in the CVVHF and EDHF groups died during the 60 days of the study period.

Some randomized controlled trials have compared the use of continuous venovenous hemodiafiltration (CVVHDF) or CVVHF with the use of IHD or sustained low-efficiency dialysis (SLED) for the treatment of AKI [10-12,17]. Collectively, these studies failed to demonstrate improved survival or renal recovery in patients on CVVHDF or CVVHF. There was also considerable variation in patient selection and RRT modalities. Mehta *et al*. [18] randomized 166 critically ill patients with severe AKI to either CRRT or IHD therapy. There was a significantly higher ICU mortality rate in subjects randomized to CVVHF (60% versus 42%; *P* = 0.02). After adjustment for severity of illness, the increased level of risk attributed to CRRT was no longer statistically significant. This is because there was a clear difference in illness severity in patients randomized to CVVHF. In our study, although patients had a lower MAP and pH prior to CVVHF, the two groups were well-balanced in terms of demographics, bacteriology, physiology, and illness severity. It is possible that the patients with hemodynamic instability and severe acidosis at time of initiation of RRT tended to receive CVVHF. After Cox regression analysis, however, neither pre-RRT MAP, nor pH were predictive of renal recovery or mortality, consistent with previous reports [19].

As septic AKI occurred frequently in the context of multi-organ dysfunction, positive fluid balances have been associated with adverse outcomes in critically ill patients. However, removal of the ultrafiltrate requires maintenance of a net neutral fluid balance. During short sessions of RRT, higher volume removal can precipitate hypotension, which increases the risk of recurrent renal injury and nonrecovery of renal function [20,21]. In the study, CVVHF patients achieved a higher volume removal per day, whereas ultrafiltration per hour was maintained at a lower level in contrast to EDHF patients. Accordingly, a negative fluid balance was present in the CVVHF group, whereas there were no differences in MAP or in the incidence of hypotension between groups in the first 72 hours of RRT. Thus, persistent and slow CVVHF therapy may be desirable for kidney recovery by removing redundant fluid that can lead to deterioration in renal function and by conferring greater hemodynamic stability to avoid further ischemic insult to the susceptible kidney [22,23].

A large observational study of sepsis secondary to pneumonia found that the highest risk of death was in patients with increased activation of inflammatory cytokines [9]. For many years, RRT has been proposed as a possible strategy to modulate the multiple inflammatory mediators [24]. Using a rodent model of cecal ligation puncture (CLP)-induced sepsis, Peng *et al*. [25] found that extracorporeal blood purification (EBP) attenuated late peaks of inflammatory mediators (for example, high-mobility group box-1 protein), improved organ (liver and renal) function, and improved long-term survival. It has been purported that adapting time and doses of RRT results in continuous removal of inflammatory mediators released incessantly in sepsis, which are largely responsible for AKI [2,3,7]. Whether or not the increased renal recovery in our patients on CVVHF therapy is secondary to mitigate the inflammatory responses that are seen in sepsis, however, needs further clarification by solid experimental and clinical evidence.

Whether or not replacement fluid at a high effluent rate improves outcome remains controversial. Two large multicenter clinical trials, the Veterans Affairs/National Institutes of Health Acute Renal Failure Trial Network (ATN) study and the Randomized Evaluation of Normal versus Augmented Level replacement therapy (RENAL) trial, randomly assigned critically ill adults with AKI requiring RRT to high-intensity or low-intensity treatment, and revealed that increasing intensity of RRT beyond conventionally recommended doses does not improve patient survival [11,26]. In a prospective randomized controlled study, however, Ronco *et al*. found that a rate of 35 ml/kg per hour had a survival benefit compared with 20 ml/kg per hour [27]. As the intensity of RRT changes occurred in both groups throughout treatment, we were unable to firmly establish a link between the replacement fluid dose and improvement in renal recovery and mortality.

Several observational studies suggest that early renal recovery may result in improved short-term mortality. These studies used mostly ICU discharge or a shorter period of hospitalization (15 days) as a primary endpoint [28,29]. During the study, we unexpectedly found that 7.69% and 7.50% patients in the CVVHF and EDHF groups died during the study period, even though they recovered renal function. This is likely because multiple factors result in an increased mortality risk in critically ill patients, including preexisting comorbidities, illness severity, and timeliness of RRT, differences in clinician approach, and patient socioeconomic status.

Our finding that severity of illness, as measured by SOFA and APACHE II scores, was an independent predictor of mortality and renal recovery on RRT is not new or unexpected [30,31]. However, our finding that CVVHF was an independent predictor of renal recovery is new and of interest. Of note, clinically relevant covariates that impact renal recovery were analyzed, and CVVHF therapy strongly correlated with improved renal recovery. Remarkably, in a recent large matched cohort study of critically ill adults with AKI requiring dialysis, Wald *et al*. [22] found that the initiation of CRRT is associated with a lower likelihood of chronic dialysis compared with IHD. In addition, a meta-analysis containing 50 studies on dialysis dependence after RRT for AKI showed that initial treatment with CRRT was associated with lower rates of dialysis dependence than intermittent renal replacement therapy (IRRT) among AKI survivors [32].

Hypophosphatemia, the adverse event related to RRT, occurred in patients on CVVHF therapy. Sodium glycerophosphate was usually injected to correct hypophosphatemia and maintain serum phosphorus levels at the standard level, so we did not record clinical complications related to hypophosphatemia. As a continuous extracorporeal therapy, CRRT frequently requires continuous anticoagulation, which increases the bleeding risk. We did not note a significant difference between groups with regards to bleeding.

Our study had several limitations. First, even through our study cohort was prospectively obtained, and data were collected from medical records and dialysis flow sheets with a presumed high level of accuracy, our study has a retrospective observational design with its inherent biases. To minimize bias, we included all critically ill patients on bedside RRT for the specified investigation period. Second, we did not capture the precise records of adjusting vasopressor drug doses during RRT, so these data are not available for comparison purposes. Third, the assertion that CVVHF therapy is superior to EDHF therapy for septic AKI cannot be translated into clinically important gains because of our small sample size. Whether continuous therapy will lead to improved prognosis in patients with septic AKI may require additional analysis with appropriately powered, designed, and conducted studies.

## Conclusion

In summary, patients with septic AKI undergoing CVVHF had an increasing incidence of and shorter time to renal recovery when undergoing CVVHF compared to EDHF, without a significant difference in 60-day mortality. Patients on CVVHF had stable hemodynamics, even in patients with lower MAP prior to initiation of treatment and negative fluid balance during treatment. The initial treatment time for CVVHF and EDHF in our study cohort reflects clinical practice, and indicates that continuous treatment is beneficial for hemodynamically unstable patients in the acute stages of septic AKI.

## Key messages

• Septic AKI patients undergoing CVVHF had a higher proportion of and shorter time to renal recovery.

• Mortality did not differ in septic AKI patients treated with either CVVHF or EDHF.

• Apart from hypophosphatemia, there were no differences in the occurrence of adverse events between the CVVHF and EDHF groups.

• Patients on CVVHF therapy had an overall negative fluid balance with more stable hemodynamics.

## Abbreviations

AKI: acute kidney injury; APACHE II: acute physiologic and chronic health evaluation II; CRP: C-reactive protein; CRRT: continuous renal replacement therapy; CVVHDF: continuous venovenous hemodiafiltration; CVVHF: continuous venovenous hemofiltration; EBP: extracorporeal blood purification; EDHF: extended daily hemofiltration; eGFR: estimated glomerular filtration rate; FB: fluid balance; HR: hazard ratio; IHD: intermittent hemodialysis; MAP: mean arterial pressure; RIFLE: risk injury failure loss end-stage renal failure; RRT: renal replacement therapy; SEM: standard error of the mean; SLED: sustained low-efficiency dialysis; SOFA: sequential organ failure assessment; UO: urine output.

## Competing interests

The authors have no competing interests to declare.

## Authors’ contributions

ZS participated in the design of the study, collected data, made the figures and table, analyzed and interpreted the data, drafted the manuscript, and revised the manuscript critically for important intellectual content. HY, XS, and HC participated in the design of the study, and analyzed and interpreted the data, drafted the manuscript with particular focus on the material and methods section. XW participated in the design of the study, helped to perform the statistical analysis, and revised the manuscript critically for important intellectual content. JY conceived the study, drafted the manuscript, participated in coordination, and revised the manuscript critically for important intellectual content. All authors have given final approval of the version to be published.
